# Coping with diabetes: Provider attributes that influence type 2 diabetes adherence

**DOI:** 10.1371/journal.pone.0214713

**Published:** 2019-04-02

**Authors:** Yolonda Freeman-Hildreth, David Aron, Philip A. Cola, Yunmei Wang

**Affiliations:** 1 College of Health Professions, University of Detroit-Mercy, Detroit, Michigan, United States; 2 Department of Medicine, Case Western Reserve University, Louis Stokes VA Medical Center, Cleveland, Ohio, United States; 3 Department of Design and Innovation, Weatherhead School of Management, Case Western Reserve University, Cleveland, Ohio, United States; 4 Department of Medicine, Case Western Reserve University, Cleveland, Ohio, United States; Florida International University Herbert Wertheim College of Medicine, UNITED STATES

## Abstract

Diabetes, a chronic disease affecting over 29 million people in the United States, requires the integration of complex medical tasks into a person’s daily life. Patient-centered care and compassion are recognized as essential dimensions of the quality care experience. This research examined provider attributes that influence adherence to type 2 diabetes mellitus (T2DM) regimens and sought to understand the phenomena of provider attributes, treatment adherence, and their relationship to coping ability and treatment outcomes. This quantitative study sampled 474 people with T2DM using a 62-item online survey administered to three different groups. The sample population included people over age 18 diagnosed with T2DM. The first group included 91 persons with T2DM identified through a Facebook group and personal social media connections, the second group included 120 Amazon Mechanical Turk participants with T2DM, and the third group included 263 respondents from a Qualtrics panel who had T2DM. Results indicated that perceived provider compassion (β = .41, ρ < .001) and optimism (β = .48, ρ < .001) positively affected coping ability. Additionally, full mediation effects for self-management were revealed, with coping ability positively mediating the effect of compassion on self-management and the effects of optimism on self-management. Furthermore, full mediation effects were found for treatment satisfaction, with coping ability positively mediating the effect of compassion on treatment satisfaction and the effects of optimism on treatment satisfaction. This research has implications for patients, healthcare professionals, and leaders suggesting that providers who communicate with optimism and compassion positively affect coping ability. As a result, healthcare providers and professionals have an opportunity to enhance self-management adherence by helping their patients cope with the burdens of diabetes. In addition, this study has implications for developing provider communication tools aimed at assessing patients’ coping capacity and increasing compassionate communication.

## Introduction

Diabetes is a chronic disease that affects over 29 million people in the United States [1]. The number of people diagnosed with diabetes increased fourfold in the United States between 1980 and 2014, with type 2 diabetes mellitus (T2DM) making up the majority of newly diagnosed cases [2]. Managing diabetes requires integrating complex medical tasks into a person’s daily life, imposing substantial psychological and behavioral demands on patients and their families. Since 2005, over 40 new drugs, including combination medications, have been approved to treat T2DM.

Despite recent developments in effective diabetes treatment and interventions to prevent diabetes complications and adverse events [3], adherence remains suboptimal [4,5], resulting in a gap between the recommended T2DM treatments and what patients actually adhere to [6,7]. Suboptimal medication adherence places T2DM patients at increased risk of preventable health complications [8–10] such as kidney disease, blindness, heart disease, and cerebral vascular disease [11], increasing their use of healthcare services and their morbidity, and mortality [12,10]. As a result of this recognized problem, there is a need to examine additional approaches that will inspire long-term adherence and glycemic control [2].

Our earlier qualitative study examined 30 African American patients with T2DM [13]. We found that T2DM adherence was influenced by positive feedback mechanisms occurring from internal or external motivational factors resulting from supportive interactions, social relationships, or the person’s ability to cope with situations. In response to the problem of continued suboptimal medication adherence, one study [11] called for additional research to investigate modifiable factors that influence adherence, targeting interventions that promote adherence, diabetes control, and reduced disease progression.

Recently, many healthcare systems have placed a greater emphasis on providing patient-centered care (PCC). PCC is respectful and preferential care that aligns with patients’ personal values in decision making [14], yielding increased satisfaction with care and improved clinical and behavioral outcomes [15]. Compassion is often referenced as a hallmark of quality care by patients, providers, health care administrators, and policy makers [16]. One study [17] found that compassionate care promotes a sense of belonging, attachment, and security, leading to bond formation. Another study [18] contended that healthcare delivery through PCC prioritizes compassion as a key factor for patient engagement and modifying health behaviors. However, studies have shown differences in patients' and providers' perceptions of compassionate care [16]. Patients today are interested in receiving health information, serving as partners, and acting as collaborators in their treatment plan, which requires providers to understand their patients’ preferences and perspectives, and consider their prior knowledge, skills, and experiences [14].

The purpose of this research is to expand on our qualitative study using quantitative methods to examine the provider attributes that influence adherence to T2DM treatment. To better understand the phenomenon of provider attributes, treatment adherence, and their relationship to coping ability and treatment outcomes, the study is guided by the following research question: Which provider attributes influence patients with T2DM to adhere to their treatment plan and have successful outcomes?

This research contributes to healthcare professionals’ deeper understanding of patient perspectives and preferences regarding their involvement in personal care, providing clinically relevant information. In addition, this research contributes to the development of personalized interventions that will address treatment adherence from patients’ perspective by recognizing their views on diabetes care. Furthermore, this study has implications for improving providers’ communication practices with their T2DM patients which may result in improved outcomes through adherence.

## Material and methods

### Theoretical development and hypotheses

While health behavioral research has predominately focused on a few theories including the Health Belief Model [19], Social Cognitive Theory [20], Theory of Reasoned Action [21], Theory of Planned Behavior [22], and the Transtheoretical Model [23], broader approaches are needed to understand health behavior and health behavioral change. Exploring new theories will allow for the construction of a more holistic theory for health behavior change than currently exists. While one theory may provide an understanding of how to motivate a person to adapt to behavioral change, another theory may provide additional insight into how to maintain behavioral change over the long-term [24].

Our theoretical foundation comprises the integration of a two-part, interconnected process that occurs throughout the patient–provider encounter. The first part of the process involves the formation of the patient–physician relationship, and is based on the path-model theory, and the second part involves a transformational process, which the patient accepts a new identity with T2DM, allowing for effective coping ability and establishing motivation for adherence to treatment.

Traditional views of the patient–provider relationship, such as the paternalistic model, emphasize the physician’s dominance by encouraging patients to consent to the advised course of treatment presented by the physician [25]. In this model, the patient lacks autonomy to make decisions or is not given the opportunity to share in the decision-making process. Over the years, more patients have begun advocating for empowerment and for autonomy over their healthcare, resulting in changes in the patient–physician relationship [25]. However, in many encounters patients are still guided toward a traditional model of care delivery, and opportunity for collaboration is limited [26]. This suggests that providers remain in a position of authority, limiting patients’ opportunities for shared understandings [26]. As a result, the need for a collaborative process has emerged, involving the communication of information between provider and patient that allows patients greater ability to make informed decisions, based on their values.

Research has linked a positive patient–provider relationship with improved treatment adherence [27], communication, and outcomes [28]. This relationship gives patients the necessary support and tools to have successful disease outcomes. Studies [28,29,30] have suggested that positive patient–provider relationships influence adaptation through mechanisms of increased support and improved communication with the provider.

#### Path-model theory

Extending theory on leadership behavior, House’s path-model theory [31] proposes that leadership behavior should enhance subordinates’ motivation, performance, and satisfaction within the organization. We contend that this relates to our setting because physicians are viewed as leaders who want to influence their patients’ behavior. This theory emphasizes that optimal patient performance is achieved when physicians modify their behavior and act to increase motivation and performance in their patients [31]. It describes three types of motivational leadership behaviors that act as independent variables: (1) directive path-goal clarifying leader behavior, which ensures that knowledge is communicated in understandable terms, appropriate guidance regarding next steps is received, and mutual expectations are acknowledged; (2) supportive leader behavior, which consists of recognizing patients’ feelings and concerns about diagnosis and treatment decisions, and allowing for shared decision making; and (3) participative leader behavior, which motivates and encourages patients throughout the illness, resulting in improved outcomes [32]. We propose that healthcare providers who modify their behaviors to emphasize a greater path-goal-directed, supportive, and participative approach will enhance their patients’ motivation by reducing obstacles and clarifying the path towards satisfaction, and self-management adherence outcomes.

#### Coping with change

The first part of this process strengthens the physician–patient relationship (path-model theory) by focusing on the provider’s ability to modify patients’ behaviors to motivate, satisfy, and improve treatment adherence [32]. As the provider–patient relationship continues to strengthen, providers should leverage this relationship to help the patient through the process of accepting their new identity of living with diabetes, establishing purpose, motivation, and effective coping strategies, thereby increasing the likelihood of adherence and treatment satisfaction. In the second part of the framework, the patient learns effective mechanisms for coping with the burdens of diabetes with the provider’s assistance. With this support, the patient learns to cope with the complexities of the diabetes disease process through strategic processes such as meaning making, learning new capabilities and skills, and increasing adaptability [33,34].

#### Hope theory

Patients with chronic illness experience varying degrees of hopefulness throughout their illness. It has been suggested that a person’s defined goals become mental targets that serve as an anchor for purposive behavior [35]. Here, hope is defined as a set of cognitive components composed of two interrelated components derived from a person’s sense of successful agency (goal-directed determination) and pathways (purposeful planning to meet desired goals) [35].

Hope theory combines the two interrelated components of pathway and agency into goal-directing thinking at various points in the temporal sequence while addressing positive and negative emotions [36]. It has been postulated that the combination of agency and pathway results in successful goal achievement if the patient believes the goal is achievable and takes steps to meet the goal [35].

However, if either cognitive component is lacking, the patient is unlikely to reach the goal [35]. In other words, patients achieve agency by believing they are capable of self-management adherence, which can be influenced through optimistic and compassionate communication from providers. The second component, pathway, is achieved when patients act to achieve adherence. This component is influenced by the patient’s ability to use coping measures that increase the probability of long-term adherence, such as problem solving, skill enhancement, finding meaning, and greater use of social support. The components of hope theory represent goal-directed thought processes theoretically central to establishing meaning [35]. The pathway component is achieved with the assistance of the provider. We propose that the components of pathway and agency will contribute to the T2DM treatment adherence process.

#### Concept model constructs

**Fig 1:**
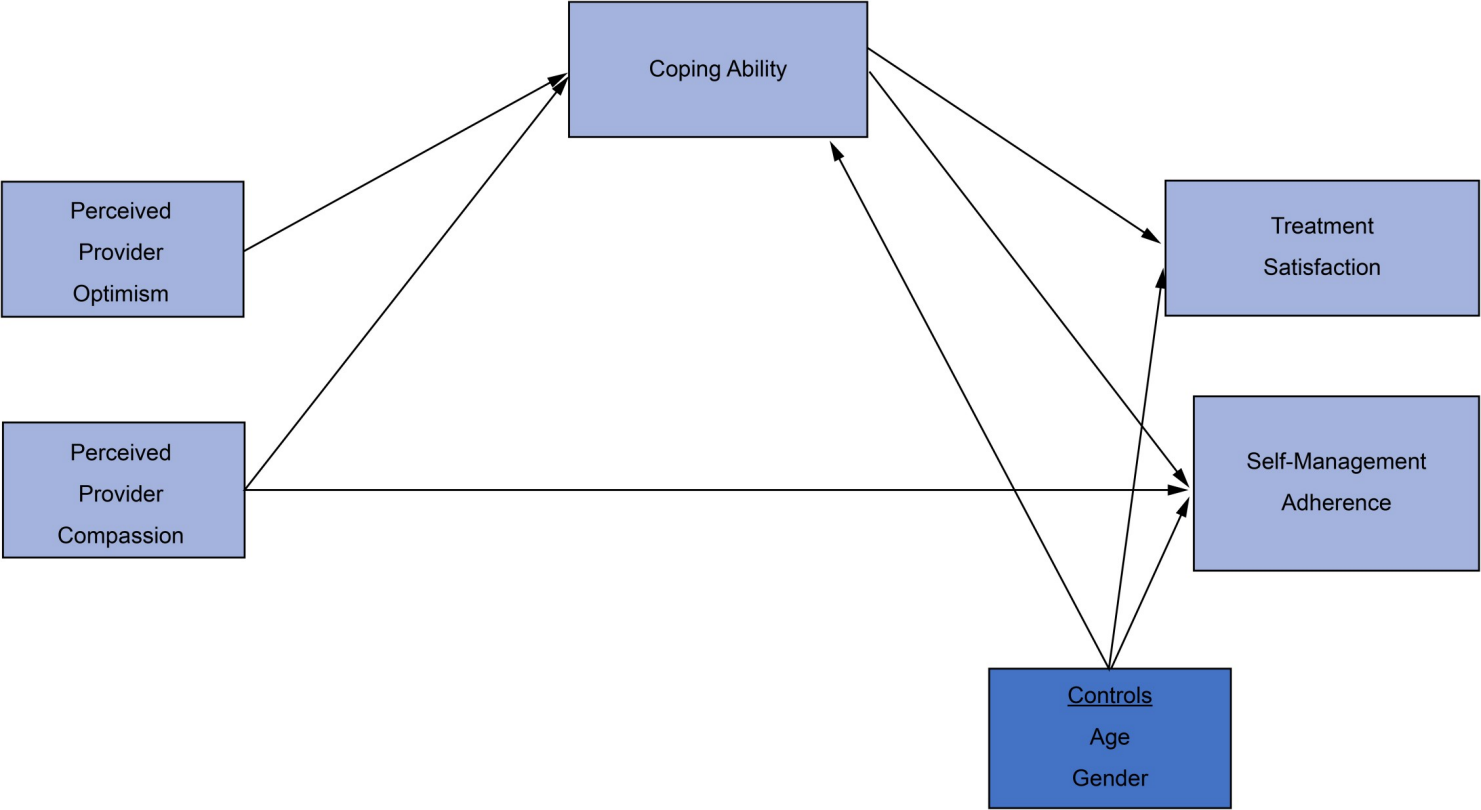
Hypothesized model.

The hypothesized model below displays each construct to be observed in our study. The independent variables (IVs) are compassion and optimism. The dependent variables (DVs) related to T2DM treatment adherence are self-management and patient treatment satisfaction. The mediating variable is coping ability, which refers to the patient’s ability to pursue goals despite setbacks, to find meaning, to find purpose, and to use social support, thereby increasing treatment adherence. See Fig 1: Hypothesized model.

In this study, we examined provider attributes for their influence on T2DM treatment adherence. Compassion is defined as patients’ perception that their concerns are respected, understood, and cared for by their provider [37]. Optimism is defined as patients’ perception of their provider as having a positive outlook.

We examined T2DM treatment adherence among our participants by evaluating two DVs. The first, self-management adherence, refers to the tasks the patient must carry out to control or reduce the impact of diabetes on their health status and daily living [38,39]. The second DV, patient treatment satisfaction, refers to the patient’s perception of satisfaction with the management and treatment received for their diabetes. In this concept model, coping ability was the mediator variable. Coping ability refers to the patient’s ability to stay motivated to achieve long-term glycemic control despite having potential threats and stressors. We anticipated that coping ability would partially mediate the provider’s intrapersonal attributes and the patient’s adherence to the treatment plan.

#### Relationship between compassion and adherence to treatment plan

When providers administer compassionate care, they connect to the patient through listening attentively, showing concern, and demonstrating an understanding of the patient’s perspective [40]. Additional relational considerations of compassionate care include “being with” patients, connecting on a human level, and taking into account the patient’s individual needs [41]. For the context of this study, compassion was the patient’s perspective of the provider’s ability to address his or her suffering or needs through relational understanding and action [16].

According to [42], compassion is a cognitive-affective behavioral process consisting of five elements: the provider’s ability to recognize suffering in others, to experience feelings of empathy, and to remain open to and accepting of another’s suffering, and the provider’s motivation to act to alleviate the suffering of others. Although compassion is closely related to empathy, there is a distinction between them. Empathy occurs when providers are able to experience their patients’ positive or negative feelings [41], as this is a necessary component for compassion, to understand and feel with another. However, when providers have compassion for their patients, they not only have empathy but they also recognize their patients’ suffering and are motivated to act to relieve the suffering.

Delivering compassionate care is essential to PCC, and leads to greater patient–provider communication, increased patient participation in treatment decisions, increased communication of contextualized knowledge, and enhanced provider emotional support [42]. When patients receive compassionate care from their providers, they are more comfortable sharing their feelings and concerns, which increases their desire to take an active role in decision making [40]. Patients with T2DM face self-care demands requiring complex adjustments around work, social, and family life, leaving many feeling defeated and unmotivated to adhere to the diabetes care regimen [43]. One study [44] proposed that compassionate care improves feelings of social connectedness, well-being, and patient satisfaction. Providers who communicate with compassion by considering their patient’s feelings or concerns while providing quality care will experience greater patient adherence [44].

We thus hypothesized the following:

Hypothesis 1. Provider compassion will positively affect adherence to self-management behaviors.

#### Coping ability as a mediator influencing adherence to self-management

Psychosocial adaptation achieved through coping mechanisms results in improved quality of life and treatment adherence for patients with diabetes, whereas lower coping ability results in greater denial, treatment dissatisfaction, depression, and psychological distress [45]. It has been suggested that greater coping ability results in positive reframing, acceptance, and problem-focused thinking, enabling patients to take responsibility for day-to-day self-management of their illness [46]. This improved coping ability results in greater confidence in managing difficulties, creating a renewed purpose and meaning association [47]. Compassionate providers develop greater connections with their patients and can better assess coping ability and motivate their continued focus on desired goals [37]. In addition, in our qualitative study [13], we found increased adherence among patients whose providers showed compassion and maintained a positive outlook.

According to hope theory, a patient’s perceived capacity to achieve a desired goal or adherence depends on a pathway and agency. In other words, patients need to believe that the goal is achievable. This can occur through acquired feelings of compassion and optimism received from provider support. It has been suggested that compassion evokes feelings of engagement, bonding, positive emotions, and emotional contagion that benefit the receiver as well as the person offering the compassion [17]. Second, the patient needs a pathway to take actions toward meeting a desired goal. Coping enables patients to positively reframe the problem, associate purpose and meaning, which results in patients changing their perspective towards their diabetes. Consequently, they adapt to change while maintaining their ability to cope with setbacks without losing their capacity to achieve their goal. We thus hypothesized the following:

Hypothesis 2a. Provider compassion and optimism will positively affect coping ability.
Hypothesis 2b. Coping ability will positively affect adherence to self-management.
Hypothesis 2c. Coping ability will partially mediate the positive relationship between compassion and adherence to self-management behaviors.
Hypothesis 2d. Coping ability will partially mediate the positive relationship between optimism and adherence to self-management behaviors.

#### Coping ability as a mediator influencing treatment satisfaction

Compassionate communication results in greater patient relationship satisfaction; however, when patients lack the opportunity to communicate feelings and concerns, they have fewer opportunities to receive validation or to gain perspectives on their care, which leads to their disengagement [45]. As patient disengagement increases, patients experience greater dissatisfaction with care. A positive state of mind is associated with improved health and successful coping [48]).

Optimism is a generalized belief in positive outcomes based on an individual’s rational estimates of the likelihood of success and belief [49]. Patients whose providers have an optimistic disposition experience increased positive emotions, connectivity, and decreased psychological distress [50], which in turn increases their capacity to cope. Effective coping capacity also increases patients’ ability to adapt to stressors through the mechanisms of problem solving, acceptance, finding meaning, seeking support, planning, and positive reframing, resulting in lower psychological distress and greater treatment satisfaction [45]. These strategies allow patients to associate meaning with the illness; even though the situation is unchanged, patients are able to find their motivation [50]. This improves their future outlook, increases their confidence toward managing illness difficulties, creates a renewed purpose, and helps them adapt to setbacks [47]. One meta-analysis [47] identified an optimistic view as a significant predictor of improved health and clinical outcomes. We, therefore, proposed the following hypotheses:

Hypothesis 3a. Coping ability will positively affect treatment satisfaction.
Hypothesis 3b. Coping ability will partially mediate the positive relationship between compassion and treatment satisfaction.
Hypothesis 3c. Coping ability will partially mediate the positive relationship between optimism and treatment satisfaction.

## Materials and methods

### Data sample and procedures

The Case Western Reserve University Institutional Review Board reviewed and approved this study. We conducted a quantitative study to test our hypotheses and validate our proposed conceptual model with a 62-item online structured survey applicable to people with T2DM. This survey was administered to investigate and collect data on the patient perspective on provider attributes that influence T2DM treatment adherence. Participants in this study self-identified as T2DM. We designed the survey with a quality control question at the beginning asking participants to certify they have Type 2 diabetes. If the participant responded “no” indicating they do not have T2DM, the survey automatically ended, and the participant was not allowed to continue.

We sampled a total of 474 people with T2DM over the age of 18 from three separate groups who self-selected to participate in the study. Excluded from this study were people diagnosed with type 1 diabetes or gestational diabetes, or those under the age of 18. Our first group consisted of 91 individuals with T2DM who were Facebook diabetes support group members and had social media connections. Participants were recruited from a social networking diabetes Facebook support group formed with a mission to provide support, encouragement, and information to help those with diabetes. We contacted the group administrator requesting permission to post our survey link onto their Facebook page for group members to access. Once permission was obtained, our survey was posted on the site with a description of our study’s purpose and an attached link directing members to our online Web-based survey. Our second group consisted of 120 individuals with T2DM recruited from Amazon Mechanical Turk. In addition to our survey’s quality control question, Amazon Mechanical Turk screens their participants, assigning each participant a unique ID that allows researchers to identify workers [51]. Finally, our third sample group consisted of 263 individuals with T2DM recruited from a Qualtrics panel. Like Amazon Mechanical Turk, Qualtrics assigns participants a unique ID to identify them in the study data.

We obtained additional demographic characteristics from participants including age and sex. Participants were 54 years old on average, were being treated with oral medications, and were predominantly female (59.8%). The majority of our sample were from the US with the exception of seven participants. The participants were predominately Caucasian (60.1%), followed by African American (29.0), Asian (4.2%), and Hispanic (3.5%). The largest share had had diabetes for over 10 years (33.6%), followed by 8 to 10 years (16.1%), 4 to 7 years (30.11%), and 0 to 3 years (20.1%). The majority of the participants’ HgB A1c levels over the last three months was <7.0 (40.4%) followed by 7.0–7.9 (30.7%), 8.0–8.9 (15.5%), 9.0–9.9 (5.8%), and >10.0 (7.6%). Demographic characteristics are summarized in Table 1.

**Table 1:** Sample demographic characteristics.

Demographic Sample Characteristics	Total Sample n = 474 surveyed	Qualtrics Sample n = 263 surveyed	Amazon Mechanical Turk n = 120 surveyed	Facebook/Social Connections n = 91 surveyed
**Gender:**				
Males:	172 (40.2%)	108 (41%)	60 (50%)	4 (8.9%)
Females	256 (59.8)	155 (59%)	60 (50%)	41 (91.9%)
**Age:**				
2017–1978	98 (28%)	19 (7%)	70 (58%)	9 (20%)
1977–1958	141 (33%)	78 (30%)	40 (33%)	23 (51%)
1957–1938	186 (43%)	163 (62%)	10 (8%)	13(29%)
Born before 1938	3 (.01%)	3 (1%)	0 (0%)	0 (0%)
**Number of Years with Diabetes**				
0–3 years	86 (20.1)	31 (12%)	37 (31%)	18 (40%)
4–7 years	129 (30.1%)	68 (26%)	49 (41%)	12(26.7%)
8–10 years	69 (16.1%)	48 (18%)	17 (14%)	4 (8.9%)
>10 years	144 (33.6%)	116 (44%)	17 (14%)	11(24.4%)
**Glucose control over the last three months**	433 responses			
HgB A1C >10 HgB A1C = 9.0 = 9.9 HgB A1C = 8.0–8.9 HgB A1C = 7.0–7.9 HgB A1C <7.0	33 (7.6%) 25 (5.8%) 67 (15.5%) 133 (30.7%) 175 (40.4%)	17 (6%) 15 (6%) 28 (11%) 80 (30%) 123 (47%)	4 (3%) 8 (7%) 34 (28%) 42 (35%) 32 (27%)	12 (27%) 2(4%) 3(7%) 11(24%) 17 (38%)
**Change in HgB level since HgB last checked** Increased Consistent Decreased	66 (15.2%) 237 (50%) 130 (30%)	44 (17%) 146 (56%) 73 (28%)	10 (8%) 76 (63%) 34 (28%)	12(27%) 13 (29%) 20 (44%)

46 respondents were excluded from the Facebook/social connection group because of excessive incomplete responses

#### Measures

Measurement items for our constructs were adapted from the literature and based on the findings from our qualitative study [13]. The scales for each construct are listed in S2 Appendix. All scales were reflective, chosen from validated measures, and adapted to reflect the patient’s perspective using a 5-point Likert scale with responses ranging from 1 (*strongly disagree*) to 5 (*strongly agree*). Our complete survey consisted of 62 items; however, for this analysis, we focused on 24 items adapted from five scales and additional items captured from demographic responses. The 24-items were chosen based upon our research question, hypothesized model, and theoretical framework. Each of our constructs for compassion, optimism, coping ability, treatment adherence, and treatment satisfaction were operationalized using reflective scales.

#### Q-sort

Our items were initially tested for content, discriminant, and convergent validity using the Q-sort technique (Q-sort). Q-sort is a well-established technique for quantitatively evaluating opinions and attitudes [52], to establish content, discriminant, and convergent validity before the post-data collection analysis. We conducted three rounds of Q-sorting using Qualtrics software. During each round, five to six participants with sufficient knowledge of T2DM were asked to review our survey items. Participants were asked to drag each item into the category they believed it related to. This process resulted in editing and minor wording changes in our final survey items before distribution.

### Operational definitions of variables

For the purpose of this study, the following operational definitions were utilized. See S3 Appendix.

- Self-management adherence refers to the tasks that individuals must carry out to control or reduce the impact of diabetes on their health status or daily living [38,39].
- Treatment satisfaction is the patient’s perception of satisfaction with the management and treatment received for their diabetes [53].
- Coping ability refers to the patient’s ability to stay motivated and persevere to achieve long-term glycemic control despite having potential threats and stressors [54].
- Optimism is the patient’s perception that their provider has a positive outlook [55].
- Compassion is operationalized by the patient’s perception that their concerns are respected, understood, and acted upon by their provider [56].

### Measurement of dependent variables

#### Diabetes self-management

We operationalized this measure by adapting five items from the Diabetes Self-Management Questionnaire (DSMQ) scale [57]. The DSMQ scale provides reliable and valid information on diabetes self-management care by assessing four specific self-management activities associated with glycemic control, including glucose management, dietary control, physical activity, and health-care use [57]. The five self-management survey items utilized in our study assess each of these four specific self-care activities. These items were reworded from the original DSMQ scale to pertain to the treatment of diabetes; the internal consistency alpha coefficient of this scale is 0.84 [57].

#### Patient treatment satisfaction

We operationalized our second DV by adapting five reflective items from the Diabetes Treatment Satisfaction Questionnaire (DTSQ) scale [58] to assess the satisfaction of our participants with their T2DM treatment. The questions utilized in this scale measured patients’ perception of satisfaction with the management and treatment received regarding their diabetes [53]. The reliability coefficient of the DTSQ scale is 0.79 [58].

### Measurement of mediating variable

#### Coping ability

We operationalized participants’ perceptions of their ability to cope with their diabetes with the Utrecht Proactive Coping Competence Scale [59], which was designed to assess proactive and problem-focused coping ability. Proactive coping focuses on anticipating, planning, and continued evaluation of self-management activities, which increases awareness of possible threats to self-management behaviors [54]. Six items from the original Utrecht Proactive Coping Competence Scale were reworded to pertain to the treatment of diabetes. The internal consistency alpha coefficient of this scale is 0.94 [59].

### Measurement of independent variables

#### Provider compassion

We measured patients’ perceptions of receiving compassion from providers with the adapted version of the Schwartz Center Compassionate Scale [60]. This scale measures patients’ perceptions that their concerns are respected, understood, and cared for by their provider. Five items from this scale were reworded from the original Compassionate scale to pertain to the treatment of diabetes. The internal consistency alpha coefficient of this scale is 0.97 [60].

#### Provider optimism

We used the Life Orientation Test–Revised (LOT-R) scale [61] to measure patients’ perceptions of their providers’ generalized sense of optimism. Four items from the original LOT-R scale were adapted to pertain to the treatment of diabetes. The internal consistency alpha coefficient of this scale is 0.76 [61].

### Controls

In this study, we controlled for age and gender because of their possible influence on treatment adherence and outcomes. Some previous studies [62,63] have found increased non-adherence among younger people with T2DM related to peer pressure to consume beverages and foods high in sugar content and with increased instances of denial of the disease. Others found elderly patients to be at greater risk for non-adherence, ranging from 6% to 55%, because of factors such as caregiver burden [64], impaired hearing [64], poor cognition [65], and polypharmacy [65]. On the other hand, adherence has been positively linked to female gender [66,67].

### Analytical techniques

To test our hypotheses, we began our analysis with data screening. Following this process, we conducted exploratory factor analysis (EFA) with IBM SPSS Statistics, version 24. Next, we confirmed our results with confirmatory factor analysis (CFA), and then we performed structural equation modeling with AMOS, version 24 software to test measurement and structural relationships. During our analysis, we tested for the presence of common method bias (CMB), performed multi-group analysis, and performed mediation analysis using Hayes’s [68] bootstrapping method with 5,000 estimates and 95% confidence intervals (CIs) to allow an empirical estimate of the sampling of the distribution of the indirect effect. As an ad-hoc analysis, we evaluated the Facebook/Social support, Amazon Mechanical Turk and Qualtrics participant groups with a one-way ANOVA to detect group differences. This was followed by a Tukey's Honest Significant Difference (HSD) post-hoc test to determine which sampled groups differed. The significance level was set α < .05 for all analyses.

### Data screening

We screened the data for complete survey responses, which resulted in the exclusion of 49 participants from our dataset for excessive incomplete responses [69]. This left 425 cases for the analyses.

We also screened for missing data by measure item or variable: self-management: DSMQ_1 (3), DSMQ_2 (3), DSMQ_3 (5), DSMQ_4 (4), and DSMQ_5 (3); treatment satisfaction: DTSQ_1 (3), DTSQ_2 (2), DTSQ_3 (2), DTSQ_4 (1), and DTSQ_5 (1); compassion: SCCS_1 (1), SCCS_2 (1), SCCS_3 (1), SCCS_4 (1), and SCCS_5 (1); coping: UPCC_1 (1), UPCC_2 (1), UPCC_3 (1), UPCC_4 (1), and UPCC_5 (1); gender (1); and age (1). We replaced the missing values using regression imputation based on the relationships between the other variables in the dataset [69]. We chose this method because missing data were random occurrences and accounted for less than 10% per respondent [69].

We screened the data for unengaged participant responses by checking the standard deviations and variances between responses; we found no unengaged respondents. We examined the data for normality by checking for skewness and kurtosis. We found evidence of kurtosis between our DVs, Diabetes Treatment Satisfaction_3, and Diabetes Self-Management _4 because the calculated absolute value was above the recommended threshold of 2.2 [70]. This resulted in the removal of the Diabetes Treatment Satisfaction_3 item given the noted observation of kurtosis and value of 4.86. We retained Diabetes Self-Management _4 based upon visual inspection of the histogram distribution and assessment of normality. It has been suggested that it is preferable to evaluate normality both visually and through statistical normality tests [71].

In addition, we screened for univariate outliers and influential outliers by analyzing the box plots of our categorical data revealing outliers within the demographic variable (age) group. Given the purpose of our study, to increase our knowledge of diabetes treatment adherence, we retained the outliers in the demographic (age) group because they reflected the natural general population. Next, we screened the data for multivariate normality by examining the Cook’s distance for influential outliers, which resulted in two respondents (Case ID 7 and Case ID 11) being identified as influential outliers and removed; this left us with 423 participants.

Finally, as part of the data-screening process, we evaluated our IVs for multicollinearity. According to [69], multicollinearity challenges the extent to which a variable can be explained by other variables. In this analysis, multicollinearity and variance inflation factor (VIF) statistics were found to be less than 3.0 and below the recommended threshold of 10.0 when regressed against both our DVs, diabetes self-management and treatment satisfaction [69] see Table 2.

**Table 2:** Multicollinearity of study variables.

DV: Self-Management	Collinearity Statistics	
	Tolerance	VIF
Compassion	.413	2.422
Optimism	.413	2.419
TreatSat	.925	1.081
DV: Treatment Satisfaction	Collinearity Statistics	
	Tolerance	VIF
Compassion	.416	2.402
Optimism	.399	2.508
Self-Mang	.870	1.149

### Measurement model

We conducted exploratory factor analysis (EFA) on our reflective 24-item survey using IBM SPSS Statistics, version 24, including Principal Axis Factoring with Promax rotation to analyze the relationships between variables and to examine the factor structure. The Kaiser-Meyer-Olkin statistic at 0.914 indicated that the data were appropriate for EFA. Additionally, Bartlett’s Test of Sphericity indicated significance and adequate intercorrelations at 0.00 [69]. Commonalities were observed among the constructs to be greater than 0.30, confirming the adequacy of the matrix and the constructs’ shared common variance with other items [69].

We identified a 21-item five-factor solution based on the following criteria: eigenvalues greater than 1 [72], scree test solution [72], and the percentage of total variance explained [73], which was 69.05%. Residuals were found to be 2%, less than the 5.0% threshold [69]. The pattern matrix confirmed factor convergence and discriminant validity based on primary factor loadings greater than 0.50 without cross-loadings less than 0.20 [74] (see S1 Table). Next, we confirmed the reliability and internal consistency of the factors based on Cronbach’s alpha levels above 0.70 [75] (see S1 Table). The Cronbach’s alpha levels were as follows: compassion: 0.93, optimism: 0.91, coping ability: 0.91, self-management: 0.87, and treatment satisfaction: 0.89.

Following the EFA, we constructed a confirmatory factor analysis model (CFA), using AMOS, version 24 software, based on EFA interpretations consisting of five latent factors and 21 items from a sample of 423 participants. Model-fit analysis revealed an adequate fit, χ2 = 358, df = 159, ρ = 0.00, CFI = .97, SRMR = .05, PNFI = .79, and RMSEA = .05 [76,77]. Next, we examined for convergent validity, discriminant validity, and reliability. Convergent validity was demonstrated by regression factor loadings greater than 0.5, and discriminant validity was determined based on average variance explained (AVE) values greater than average shared variance (ASV) and maximum shared variance (MSV) for each measure [69]; see Table 3 and S1 Fig). Last, we determined reliability by analyzing the composite reliability level for each factor; all were greater than the recommended 0.70 threshold (see Table 3).

**Table 3:** Validity and reliability of confirmatory factor analysis.

Construct	CR	AVE	MSV	Compassion	Self-mangt	Optimism	Coping	Treat sat
**Compassion**	0.915	0.729	0.532	**0.854**				
**Self-mangt**	0.876	0.542	0.158	0.263	**0.736**			
**Optimism**	0.915	0.73	0.57	0.714	0.322	**0.855**		
**Coping**	0.9	0.693	0.57	0.73	0.33	0.755	**0.832**	
**Treat sat**	0.883	0.791	0.158	0.221	0.398	0.223	0.283	**0.889**

Following our CFA analysis, we evaluated our model for common method bias (CMB). The occurrence of CMB has been shown to increase when using self-reported measures obtained by a single method using a single source [78]. To account for this, we added a common latent factor (CLF) to our original CFA model, forming an unrestricted model (see S2 Fig). This process allowed free estimation of regression loadings and comparison of our unrestricted model with our nested models (zero-constrained and equal-constrained). The CLF unrestricted model resulted in an appropriate model fit, χ2 = 261, df = 139, ρ = 0.00, CFI = .98, SRMR = .03, PNFI = .70, and RMSEA = .05 [76,77]. Our zero and equal constrained models were compared with our unconstrained model resulting in a significant chi-square test (p < .001) for the zero-constrained and equal-constrained models. As a result, we concluded that, to some degree, CMB exists in the original model that served as the basis for our CMB testing (see Table 4).

**Table 4:** Common method bias nested model comparison.

Assuming model unconstrained to be correct:				
Model	x2	df	delta	p-value
Model unconstrained	261.37	139		
Model zero constrained	358.00	159	20	0.00
Model equal constrained	358.00	158	19	0.00

To further account for CMB, we imputed the factor scores of our constructs with the CLF attached.

## Results

Descriptive statistics and correlations between variables are presented in Table 5. The results indicate significant bivariate relationships between self-management and optimism (r = .36, p = .01), compassion (.30, p = .01), coping (.39, p = .01) and treatment satisfaction (.44, p = .01). Additionally, we observed significant bivariate relationships between treatment satisfaction and optimism (.26, p = .01), compassion (.26, p = .01), and coping (.32, p = .01). Interestingly, self-management was negatively affected by age (−.15, p = .01) and gender (−.10, p = .05). However, we found no significance difference in treatment satisfaction by age or gender.

**Table 5:** Correlations between study variables.

Construct	Mean	Std. Dev	Compassion	Optimism	Treat-sat	Self-mangt	Coping	Gender	Age
Compassion	3.82	0.81	1.00						
Optimism	4.24	0.70	.764**	1.00					
Treat-sat	3.87	1.11	.258**	.256**	1.00				
Self-mangt	4.09	0.83	.299**	.358**	.443**	1.00			
Coping	3.71	0.65	.784**	.800**	.323**	.391**	1.00		
Gender	1.60	0.49	-0.03	0.01	-0.09	-.104*	0.00	1.00	
Age	2.17	0.84	-0.01	-0.06	0.03	-.146**	-0.01	0.07	1.00

** Correlation is significant at the 0.01 level (2-tailed)

*Correlation is significant at the 0.05 level (2-tailed)

### Structural model

In the second part of our analysis, we examined the structural relationships of our conceptual model using AMOS software version 24. Our analysis revealed an appropriate model fit, χ2 = 3.68, df = 3, ρ = 0.30, CFI = .99, SRMR = .01, PNFI = .14, and RMSEA = .02, see S3 Fig [64]. Multiple squared correlations indicated that our IVs collectively explained the variance in coping ability (71%), self-management (29%), and treatment satisfaction (11%).

### Variable path evaluation

Next, we examined significant structural relationships within our conceptual model.

Our analysis revealed coping ability to be positively correlated with self-management (β = .38, ρ < .001), compassion (β = .41, ρ < .001), and optimism (β = .48, ρ < .001). Conversely, age (β = −.15, ρ < .001) was found to be negatively correlated with treatment satisfaction. Interestingly, gender was not significantly correlated with self-management or patient treatment satisfaction. We examined alternative structural relationships by testing additional models. Our analysis showed that the addition of our control variables had no impact on either of our IVs (see Table 6 and S4 Fig, Alternative structural models).

**Table 6:** Alternative structural model: No controls and gender.

DV: self-manage-model (no controls)		DV: treat sat-model no controls		DV: self-manage- model gender		DV: treat sat model gender		DV: self-mangt model gender plus all ages		DV: treat sat-model gender plus all ages	
**Independent** **variable**	**b(SE)**	**Independent** **variable**	**b(SE)**	**Independent** **variable**	**b(SE)**	**Independent** **variable**	**b(SE)**	**Independent** **variable**	**b(SE)**	**Independent** **variable**	**b(SE)**
Compassion	.-02 (07)	Compassion	.02 (1)	Compassion	.-03(.07)	Compassion	.01(.10)	Compassion	.-03(.07)	Compassion	.01(.10)
Coping	.38(.09) ***	Coping	.54(.13) ***	Coping	.39(.09) ***	Coping	.55(.13) ***	Coping	.38(.09) ***	Coping	.55(.13) ***
				**Control variables**		**Control variables**		**Control variables**		**Control variables**	
				Gender	-.13(0.7)	Gender	-.19(.10)	Gender	-.11(.07)	Gender	-.20(.10)
								All ages	.15(.04) ***	All ages	.05(.06)

p < .05 significance,

**p < .01 significance,

***p < .001 significance

### Mediation

**Fig 2:**
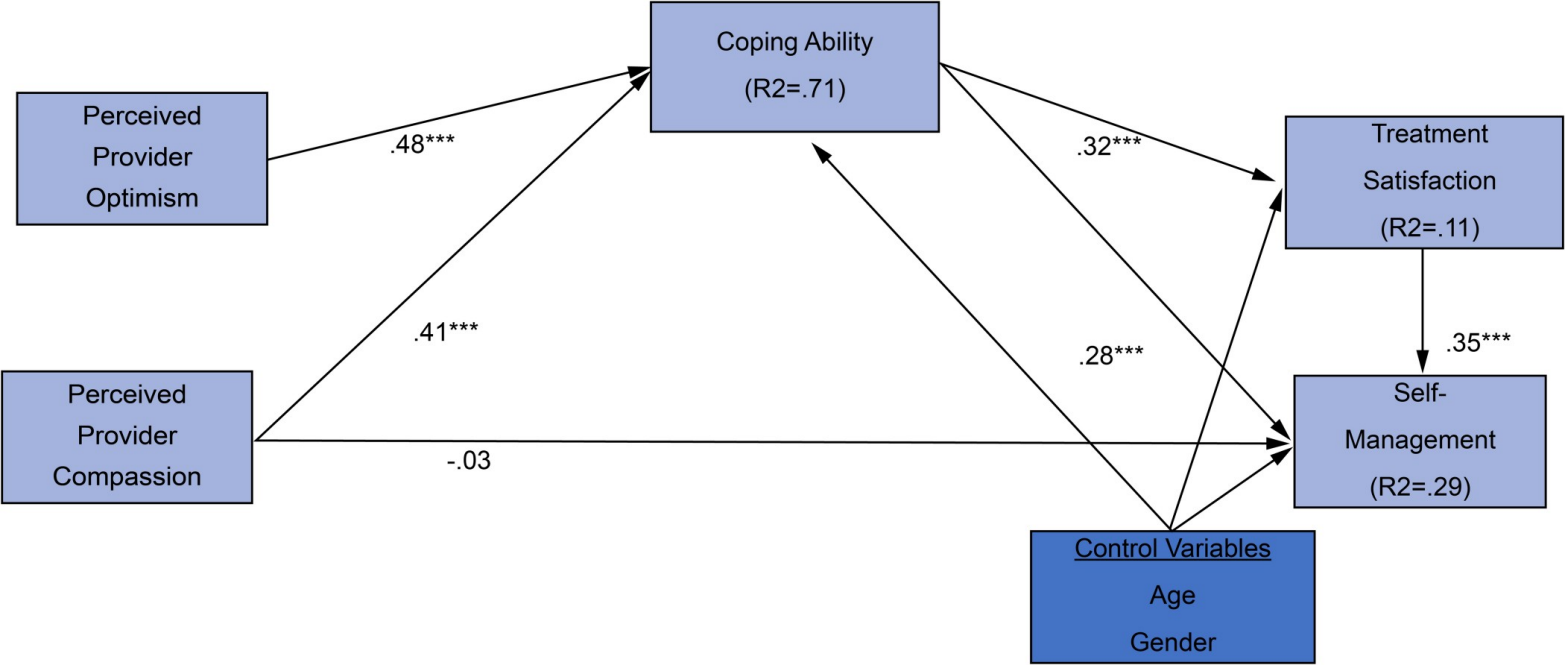
Structural model results of mediation effects. Structural Equation Modeling pathway connecting perceived provider optimism and perceived provider compassion to diabetes treatment satisfaction and self-management adherence.

We conducted mediation analysis using Hayes’s [68] method with bootstrapping using 5,000 estimates and 95% CIs to empirically estimate the sampling of the distribution of the indirect effect. We examined the mediated indirect effects using estimand programming and AMOS version 24 software [79]. Our results indicated that compassion (β = .42, ρ < .001) and optimism (β = .48, ρ < .001) were positively related to coping ability, which in turn was positively related to self-management (β = .28, ρ < .001) and treatment satisfaction (β = .32, ρ < .001), supporting Hypotheses 2c, 2d, 3b and 3c. Interestingly, compassion and optimism had no significant direct relationship with either self-management or patient satisfaction indicating a full mediation effect with our mediator, coping ability. Therefore, Hypothesis 1 was not supported (see Table 7). Of our control variables, age (β = −.15, ρ >.001) had a direct, negative relationship with self-management, and gender had no significant effect on either self-management (β = −.06, ns) or patient treatment satisfaction (β = −.09, ns). Our mediation analysis showed full mediation effects for coping ability, which positively mediated the relationship between compassion (β = .13, bias-corrected 95% CI = [.08, .19], ρ < .001) and self-management, and between provider optimism (β = .17, bias-corrected 95% CI = [.09, .27], ρ < .001) and self-management, supporting Hypotheses 2a, 2b, 2c, and 2d. Surprisingly, a full mediation effect emerged for coping ability, which mediated the effects of compassion (β = .18, bias corrected 95% CI = [.13, .25], ρ < .001) on patient treatment satisfaction and of provider optimism (β = .24, bias-corrected 95% CI = [.16, .35], ρ < .001) on patient treatment satisfaction, supporting Hypotheses 3a, 3b, and 3c (see Table 8 and Fig 2). In Table 9, we summarize our tested hypothesized relationships and results.

**Table 7:** Direct effects.

Direct effects table	Self-management	Treatment satisfaction
Compassion	β = -0.03, ρ = ns	β = -0.00, ρ = ns
Optimism	β = 0.11, ρ = ns	β = 0.00, ρ = ns

**Table 8:** Indirect effects summary.

	Mediation type	Self-management	Patient satisfaction
Compassion	Full	0.13, [CI = 08, 0.19] ***	0.18, [CI = 13, 0.25] ***
Optimism	Full	0.17, [CI = 09, 0.27] ***	0.24, [CI = 16, 0.35] ***

***p<0.001

**Table 9:** Summary of hypothesized relationships.

Hypothesis 1	Provider compassion positively affects adherence to self-management behaviors.	Not supported	β = .-.03, ρ = ns
Hypothesis 2a	Provider compassion and optimism positively affect coping ability.	Supported	β = .41, ρ = < .001(Compassion) β = .48, ρ = >.001 (Optimism)
Hypothesis 2b	Coping ability positively affects adherence to self-management.	Supported	β = .38, ρ = < .001
Hypothesis 2c	Coping ability partially mediates the positive relationship between compassion and adherence to self-management.	Supported Full Mediation	β = .13, ρ = < .001
Hypothesis 2d	Coping ability partially mediates the positive relationship between optimism and adherence to self-management.	Supported Full Mediation	β = .17, ρ = < .001
Hypothesis 3a	Coping ability positively affects adherence to treatment satisfaction.	Supported	β = .55, ρ = < .001
Hypothesis 3b	Coping ability partially mediates the positive relationship between compassion and treatment satisfaction.	Supported Full Mediation	β = .18, ρ = < .001
Hypothesis 3c	Coping ability partially mediates the positive relationship between optimism and treatment satisfaction.	Supported Full Mediation	β = .24, ρ = < .00

### Ad-hoc analysis

We conducted a one-way ANOVA between groups comparing our Facebook diabetes social support group, Amazon Mechanical Turk, and Qualtrics groups to explore the impact on self-management and treatment satisfaction. Results revealed significant group differences among the groups with p = < .05. Post-hoc analysis comparisons using the Tukey’s Honest Significant Difference test indicated that the majority of our mean scores for Group 2 (Facebook diabetes social support group) were significantly different from Group 1(Amazon Mechanical Turk) and Group 3 (Qualtrics). This indicates that Group 1 (Amazon Mechanical Turk) and Group 3 (Qualtrics) were more similar. See S3 and S4 Tables.

## Discussion

The purpose of this research was to examine the provider attributes that influence adherence to T2DM diabetes treatment. We analyzed the provider attributes compassion and optimism and our mediator variable, coping ability, to determine their influence on T2DM self-management adherence and patient treatment satisfaction. Three significant findings emerged.

First, coping ability significantly influenced T2DM self-management adherence and treatment satisfaction outcomes. Further, coping ability fully mediated the positive relationship between self-management and treatment satisfaction via provider compassion and optimism, with an R-squared of 71%. Practically, this illustrates that provider support enhances self-management adherence by helping the patient cope with the burdens of diabetes. People with T2DM have greater self-management adherence and treatment satisfaction if they have increased coping abilities. Healthcare providers have an opportunity to motivate individuals to adhere to long-term behavioral change by evaluating their patients’ intrinsic goals and coping ability. This involves evaluating the patient’s inner beliefs and values that contribute to behavioral change and provide associated meaning [80]. Once intrinsic goals are identified, providers can offer coping strategies and resources to increase opportunities for long-term adherence and successful techniques to overcome obstacles [80]. While most diabetes interventions provide support for starting behavioral change, many fail to address how to motivate people to maintain behavioral change over the long-term. Goal formation is considered central to a person’s meaning system, resulting in a greater sense of purpose and perception of significance [81]. For healthcare professionals and providers, obtaining this knowledge requires actively listening to a patient’s story, concerns, and goals while assessing the patient’s coping ability.

Second, we found no direct relationship between our IVs compassion and provider optimism and our DVs. Essentially, this demonstrates that providers lack sole influence; the patient is necessary for achieving long-term diabetes treatment adherence. These results support the superiority of a collaborative relationship between provider and patient rather than the traditional paternalist model, which relies on greater physician control and decreased patient autonomy. According to our theoretical model, diabetes adherence consists of a two-part, interconnected process entailing the patient–provider relationship and a transformational process that allows for effective coping by establishing the patient’s motivation for adhering to treatment. In this case, providers’ increased compassion and optimism will increase patients’ positive emotions, bonding, engagement, and well-being [35], thereby increasing patients’ agency or feelings of perceived capacity of goal accomplishment. In turn, having a greater coping ability, achieved with provider assistance, allows patients to pursue the goals that bring meaning and hope to their lives, thereby increasing their motivation for adherence [82].

Third, we were surprised to find significant differences self-management and treatment satisfaction outcomes across our three sample groups. We believe there is a difference in the Facebook/Social connection group because participants in this group intentionally reached out to others for needed support, encouragement, or assistance with managing their T2DM. In this situation, the Facebook/online forum provided a non-judgmental, supportive outlet for individuals with T2DM to receive the support and resources needed to cope with the burdens of T2DM. Compared to the Facebook/Social connection group, participants in the Amazon Mechanical Turk and Qualtrics groups likely had established supportive and coping mechanisms for dealing with the burdens of diabetes. Additional research should further examine the relationship between the utilization of online social networking sites and T2DM self-management behaviors.

### Limitations and implications for future research

We recognize our study has some limitations. Our participants were self-identified as T2DM. Future research should explore this phenomenon confirming the diagnosis with verified health records. Further, this study was conducted within the context of one chronic illness (diabetes). Replication of this study in the context of additional diseases will increase the generalizability of the results. Further, although we attempted to obtain multiple responses from multiple sources to rule out CMB, we could not provide different sources for the data collection of our IVs and DVs. To account for possible CMB, we imputed the factor scores with the CLF. We encourage future researchers to attempt a variety of research designs and procedures to obtain multiple responses. While this study demonstrated the effect of coping ability on improving self-management and treatment satisfaction, a longitudinal design would provide validity over time and identify patterns. Moreover, we recognize that other variables that possibly influence self-management adherence and treatment satisfaction were not included in this study, such as social support, regimen complexity, treatment type, treatment duration, and ethnicity. Further, we acknowledge that our sample groups are unevenly distributed with increased missing data noted within the Facebook/Social Connection group. Last, we identify that the ethnic distribution of our sample population does not reflect that of people with diabetes in the United States.

Nevertheless, these findings have significant implications for patients, healthcare professionals, and leaders for improving patient–provider communication and diabetes management by implementing provider assessment tools to evaluate the coping ability of T2DM patients and increase compassionate care. Also, providers have an opportunity to influence self-management adherence by assessing their patient’s ability to cope with the burdens of diabetes and developing individualized goals that exemplify the patient’s values and beliefs. Last, providers are able to influence self-management adherence by communicating with optimism and hope while conveying realistic expectations to their patients with compassion.

## Supporting information

S1 Appendix List of study variable names. (DOCX)

S2 Appendix Constructs. (DOCX)

S3 Appendix Operational definitions of variables. (DOCX)

S1 Fig Confirmatory factor analysis model. (DOCX)

S2 Fig Confirmatory factor analysis with common latent factor. (DOCX)

S3 Fig Structural model (all controls). (DOCX)

S4 Fig Alternative model (gender control). (DOCX)

S5 Fig Structural model (no controls). (DOCX)

S1 Table Pattern matrix. (DOCX)

S2 Table Factor correlation matrix. (DOCX)

S3 Table One-way ANOVA between groups. (DOCX)

S4 Table Comparison of group means. (DOCX)
