# Supplementary material for: Coping with diabetes: Provider attributes that influence type 2 diabetes adherence
Source: PLoS One. 2019 Apr 2;14(4):e0214713. doi: 10.1371/journal.pone.0214713 (PMC6445439; doi:10.1371/journal.pone.0214713)
Supplement: S1 Appendix — (DOCX) [file pone.0214713.s001.docx]

# S1 Appendix. List of study variable names

1. Compassion (IV)
2. Optimism (IV)
3. Coping Ability (M)
4. Self-Management (DV)
5. Treatment Satisfaction (DV)
6. Gender (Control)
7. Age (Control)
