# Supplementary material for: Coping with diabetes: Provider attributes that influence type 2 diabetes adherence
Source: PLoS One. 2019 Apr 2;14(4):e0214713. doi: 10.1371/journal.pone.0214713 (PMC6445439; doi:10.1371/journal.pone.0214713)
Supplement: S2 Appendix — (DOCX) [file pone.0214713.s002.docx]

# S1 Appendix

# List of study variable names

1. Compassion (IV)
2. Optimism (IV)
3. Coping Ability (M)
4. Self-Management (DV)
5. Treatment Satisfaction (DV)
6. Gender (Control)
7. Age (Control)

**S2 Appendix**

**Constructs**

| **Construct** | **Operational Definition Items Source** | | |
| --- | --- | --- | --- |
| **Compassion (IV)** | The patient’s perception that their concerns are respected, understood, and acted upon by their provider. | **The Schwartz Center**  **Compassionate Scale**  Scoring: 1=Strongly disagree  2=Disagree 3=Neutral 4=Agree 5=  Strongly Agree  My provider, physician assistant, or  nurse practitioner . . .    1.listens to my concerns about my diabetes.  2.shows concern about my feelings.  3. respects my feelings about my diabetes treatment plan.  4.cares about my feelings I have regarding my diabetes treatment.  5.shows concern for my well-being. | Lown, B. A., Muncer, S. J., & Chadwick, R. (2015). Can compassionate healthcare be measured? The Schwartz Center  Compassionate Care Scale. Patient education and counseling, 98(8),  1005-1010.  Cronbach alpha is 0.97 |
| **Optimism (IV)** | The patient’s perception that their provider has a positive outlook. | **The Revised Life Orientation Scale**  Scoring: 1=Strongly Disagree 2=Disagree 3= I neither agree or disagree 4=Agree 5= Strongly Agree  1. In uncertain times, my provider remains positive about my diabetes.  2. My provider is optimistic about my future with diabetes.  3. My provider is optimistic my diabetes will improve with management.  4. My provider remains optimistic that we can make a difference with my diabetes. | Scheier, M. F., Carver, C. S., & Bridges, M. W. (1994). Distinguishing optimism from neuroticism (and trait anxiety, self-mastery, and self-esteem): a reevaluation of the Life Orientation Test. Journal of personality and social psychology, 67(6), 1063.  Cronbach alpha is 0.78 |

| **Treatment satisfaction (DV)** | The patient’s perception of satisfaction with the management and treatment received for their diabetes. | **Diabetes Treatment Satisfaction Questionnaire (DTSQ)**  Scoring: 1=Strongly Disagree 2=Disagree 3= I neither agree or disagree 4=Agree 5= Strongly Agree  1. I’m satisfied with my diabetes treatment.  2. I am satisfied with my diabetes control.  3. I am satisfied with my lifestyle with my diabetes treatment plan.  4. I’m satisfied with my understanding of my diabetes treatment plan.  5. I would be happy to continue with my current diabetes treatment plan. | Bradley, C., & Lewis K. (1990). Measures of psychological well-being and treatment satisfaction developed from the people with tablet-treated diabetes. Diabetic Medicine, 7(5), 445  451.  Cronbach alpha is 0.79 |
| --- | --- | --- | --- |
| **Self-Management (DV)** | The tasks that individuals must carry out to control or reduce the impact of diabetes on their health status or daily living. | **Diabetes Self-Management Questionnaire (DSMQ)**  Scoring: 1=Strongly Disagree 2=Disagree 3= I neither agree or disagree 4=Agree 5= Strongly Agree  1. I check my blood sugar as instructed by my provider.  2. I manage to keep my appointments for my diabetes care.  3. I keep track of my blood sugar levels.  4. I exercise regularly to achieve optimal blood sugar levels.  5. I keep track of my dietary intake as instructed by my provider. | Schmitt, A., Gahr, A., Hermann, N., Kulzer, B., Huber, J., & Haak, T. (2013). The Diabetes Self-Management Questionnaire (DSMQ): development and evaluation of an instrument to assess diabetes self-care activities associated with glycemic control. Health and Quality of Life Outcomes, 11(1),138.  Cronbach alpha is 0.84 |
| **Coping Ability (Mediator)** | The patient’s ability to stay motivated and persevere to achieve long-term glycemic control despite having potential threats and stressors. | **Utrecht Proactive Coping Competence Scale**  Scoring: 1=Strongly Disagree 2=Disagree 3= I neither agree or disagree 4=Agree 5= Strongly Agree  1. I consider the positive when having a setback with my diabetes.  2. I have learned from the setbacks from my diabetes.  3. I do not give up improving my health even when I have a setback.  4. I cope with my diabetes by asking others for advice when I have setbacks.  5. I can recognize when I need support to cope with problems due to my diabetes. | Tielemans, N.S., Visser-Meily, J.M., Schepers, V. P., Post., M. W., & vanHeugten, C. M. (2014). Proactive coping poststroke psychometric properties of the Utrecht Proactive Coping Competence Scale. Archives of physical medicine and rehabilitation, 95(4), 670-675.  Cronbach alpha is 0.94. |

**S1 Table. Pattern matrix**

| **Construct/Cronbach** |  | | | | |
| --- | --- | --- | --- | --- | --- |
|  | **Compassion** 0.93 | **Self-Managt** 0.88 | **Optimism**  0.91 | **Coping Ability**  0.91 | **Treatment Sat.** 0.88 |
| **SCCS_1** | 0.802 |  |  |  |  |
| **SCCS_2** | 0.741 |  |  |  |  |
| **SCCS_3** | 0.867 |  |  |  |  |
| **SCCS_4** | 0.936 |  |  |  |  |
| **SCCS_5** | 0.800 |  |  |  |  |
| **UPCC_1** |  |  | 0.937 |  |  |
| **UPCC_2** |  |  |  | 0.504 |  |
| **UPCC_3** |  |  |  | 0.746 |  |
| **UPCC_4** |  |  |  | 0.937 |  |
| **UPCC_5** |  |  |  | 0.859 |  |
| **DSMQ_1** |  | 0.648 |  |  |  |
| **DSMQ_2** |  | 0.675 |  |  |  |
| **DSMQ_3** |  | 0.803 |  |  |  |
| **DSMQ_4** |  | 0.784 |  |  |  |
| **DSMQ_5** |  | 0.759 |  |  |  |
| **LOS_2** |  |  | 0.816 |  |  |
| **LOS_3** |  |  | 0.876 |  |  |
| **LOS_4** |  |  | 0.716 |  |  |
| **DTSQ_1** |  | 0.747 |  |  |  |
| **DTSQ_2** |  |  |  |  | 0.891 |
| **DTSQ_4** |  |  |  |  | 0.885 |
| Extraction Method: Principal Axis Factoring.   Rotation Method: Promax with Kaiser Normalization. | | | | | |
| a. Rotation converged in 6 iterations. | | | | | |

**S2 Table. Factor correlation matrix**

| **Construct** | **Compassion** | **Self-Mgt** | **Optimism** | **Coping Ability** | **Treat. Sat.** |
| --- | --- | --- | --- | --- | --- |
| **Compassion** | 1.000 | 0.279 | 0.688 | 0.694 | 0.254 |
| **Self-Mgt** | 0.279 | 1.000 | 0.324 | 0.290 | 0.382 |
| **Optimism** | 0.688 | 0.324 | 1.000 | 0.696 | 0.260 |
| **Coping Ability** | 0.694 | 0.290 | 0.696 | 1.000 | 0.263 |
| **Treatment Sat.** | 0.254 | 0.382 | 0.260 | 0.263 | 1.000 |
| Extraction Method: Principal Axis Factoring.   Rotation Method: Promax with Kaiser Normalization. | | | | | |
