# Supplementary material for: Coping with diabetes: Provider attributes that influence type 2 diabetes adherence
Source: PLoS One. 2019 Apr 2;14(4):e0214713. doi: 10.1371/journal.pone.0214713 (PMC6445439; doi:10.1371/journal.pone.0214713)
Supplement: S3 Appendix — (DOCX) [file pone.0214713.s003.docx]

# **S3 Appendix. Operational definitions of variables**

For the purpose of this study, the following operational definitions were utilized.

- Self-management adherence refers to the tasks that individuals must carry out to control or reduce the impact of diabetes on their health status or daily living [38,39].
- Treatment satisfaction is the patient’s perception of satisfaction with the management and treatment received for their diabetes [53].
- Coping ability refers to the patient’s ability to stay motivated and persevere to achieve long-term glycemic control despite having potential threats and stressors [54].
- Optimism is the patient’s perception that their provider has a positive outlook [55].
- Compassion is operationalized by the patient’s perception that their concerns are respected, understood, and cared for by their provider [56].
