# Supplementary figures and images for: Coping with diabetes: Provider attributes that influence type 2 diabetes adherence
Source: PLoS One. 2019 Apr 2;14(4):e0214713. doi: 10.1371/journal.pone.0214713 (PMC6445439; doi:10.1371/journal.pone.0214713)

**S1 Fig. Confirmatory factor analysis model (standardized values)**


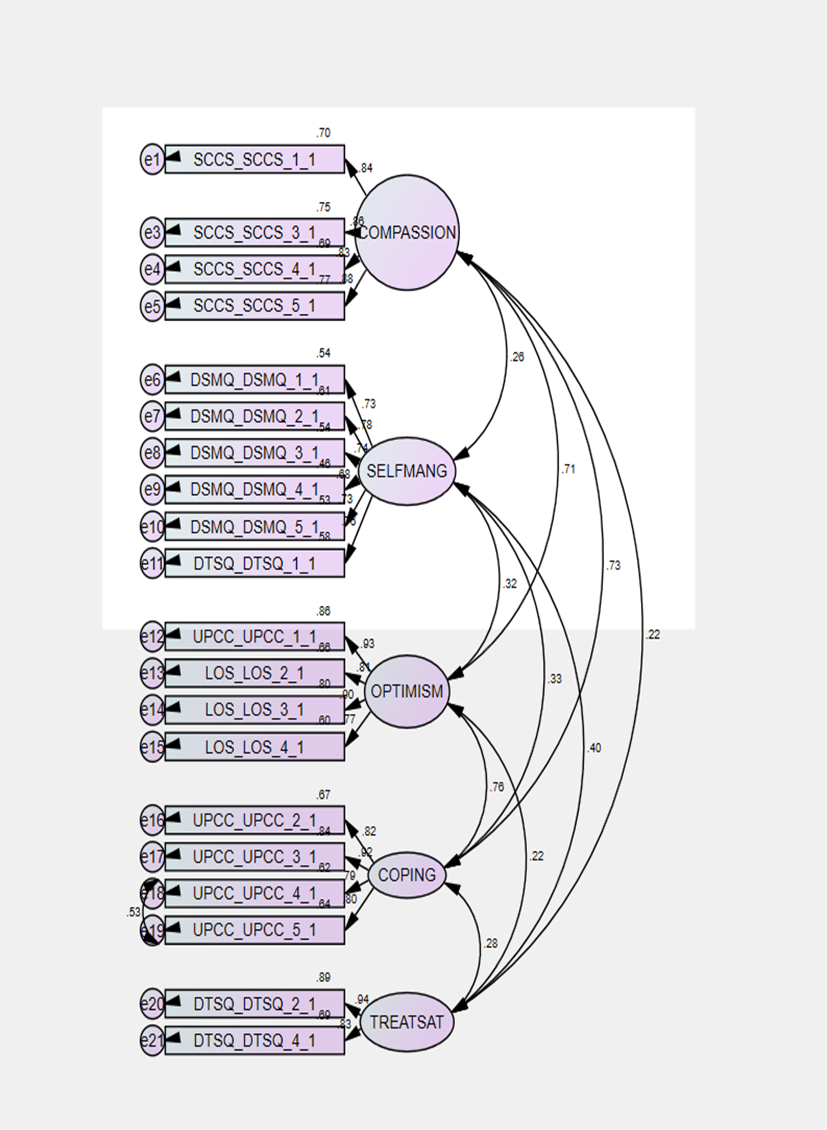

Supplement: S1 Fig — (DOCX) [file pone.0214713.s004.docx]

**S2 Fig. Confirmatory factor analysis with common latent factor (standardized values)**


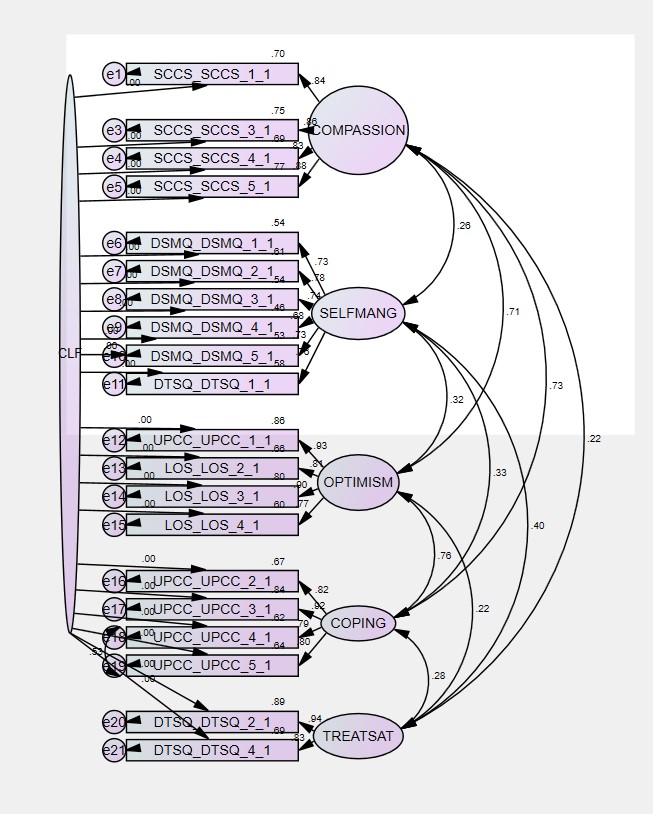

Supplement: S2 Fig — (DOCX) [file pone.0214713.s005.docx]

**S3 Fig. Structural model (all controls) standardized values**


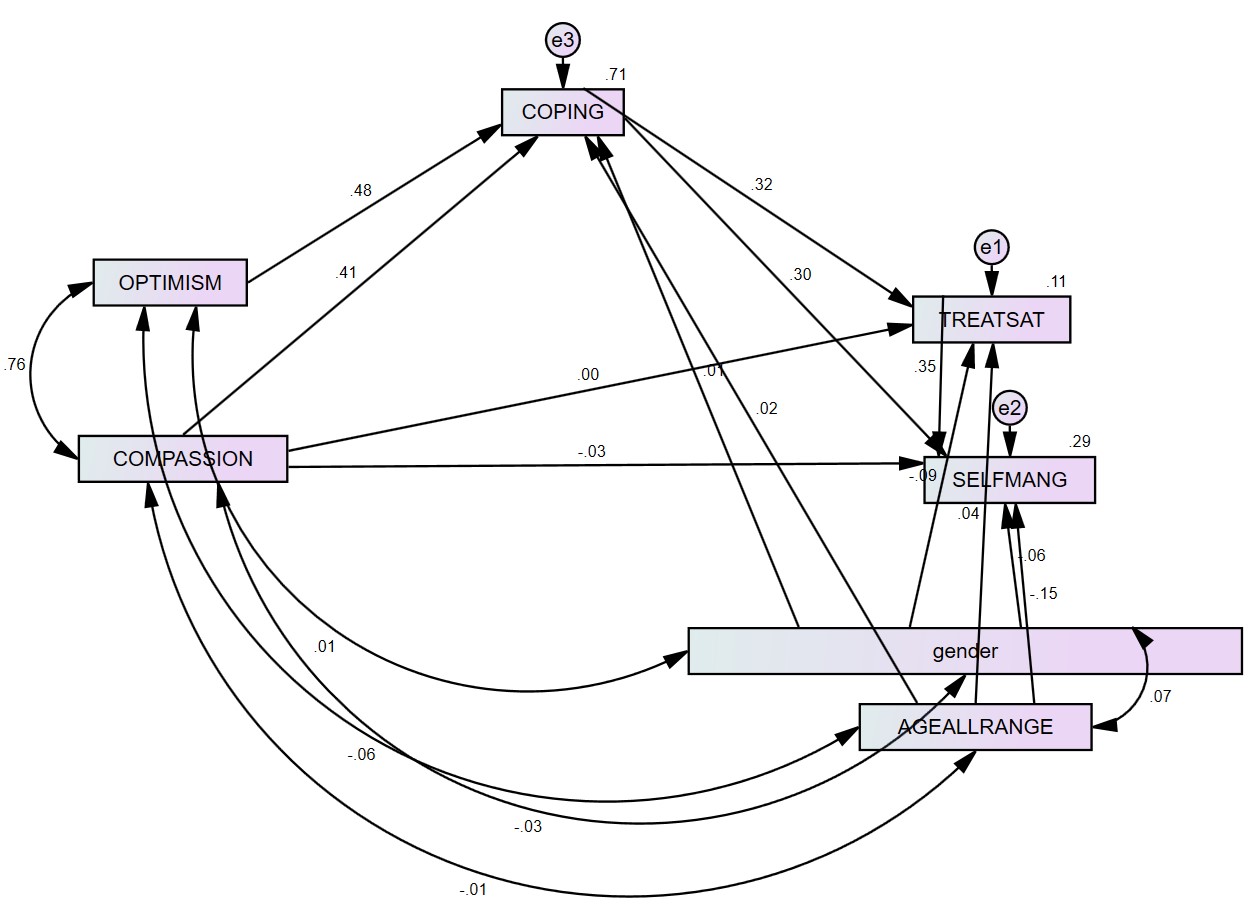

Supplement: S3 Fig — (DOCX) [file pone.0214713.s006.docx]

S4 Fig. Alternative model

W/gender control (standardized values)


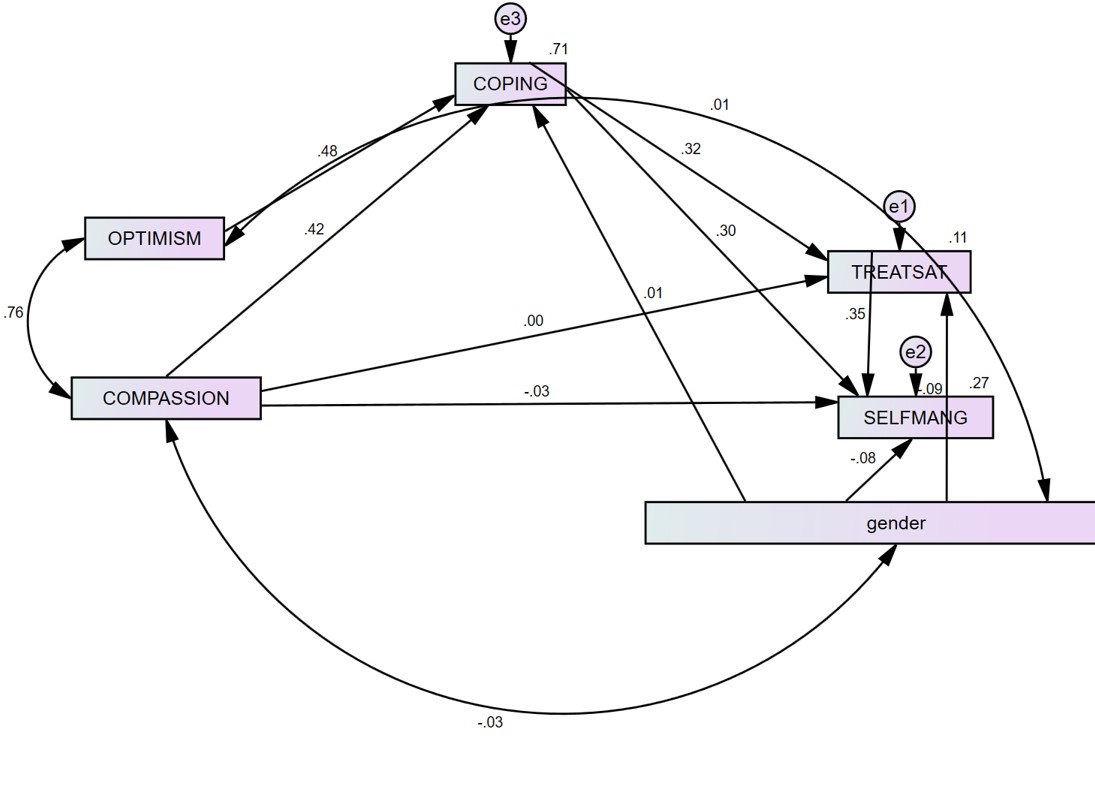

Supplement: S4 Fig — (DOCX) [file pone.0214713.s007.docx]

S5 Fig. Structural model

No controls (standardized values)


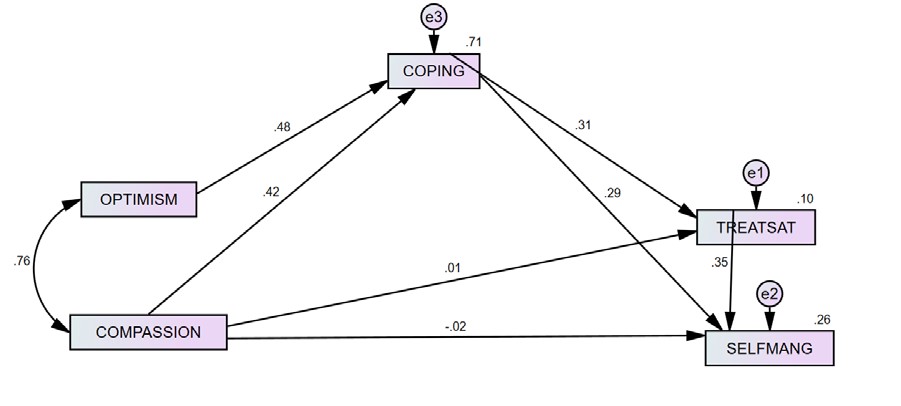

Supplement: S5 Fig — (DOCX) [file pone.0214713.s008.docx]
