# Supplementary material for: Coping with diabetes: Provider attributes that influence type 2 diabetes adherence
Source: PLoS One. 2019 Apr 2;14(4):e0214713. doi: 10.1371/journal.pone.0214713 (PMC6445439; doi:10.1371/journal.pone.0214713)
Supplement: S1 Table — (DOCX) [file pone.0214713.s009.docx]

**S1 Table. Pattern matrix**

| **Construct/Cronbach** |  | | | | |
| --- | --- | --- | --- | --- | --- |
|  | **Compassion** 0.93 | **Self-Managt** 0.88 | **Optimism**  0.91 | **Coping Ability**  0.91 | **Treatment Sat.** 0.88 |
| **SCCS_1** | 0.802 |  |  |  |  |
| **SCCS_2** | 0.741 |  |  |  |  |
| **SCCS_3** | 0.867 |  |  |  |  |
| **SCCS_4** | 0.936 |  |  |  |  |
| **SCCS_5** | 0.800 |  |  |  |  |
| **UPCC_1** |  |  | 0.937 |  |  |
| **UPCC_2** |  |  |  | 0.504 |  |
| **UPCC_3** |  |  |  | 0.746 |  |
| **UPCC_4** |  |  |  | 0.937 |  |
| **UPCC_5** |  |  |  | 0.859 |  |
| **DSMQ_1** |  | 0.648 |  |  |  |
| **DSMQ_2** |  | 0.675 |  |  |  |
| **DSMQ_3** |  | 0.803 |  |  |  |
| **DSMQ_4** |  | 0.784 |  |  |  |
| **DSMQ_5** |  | 0.759 |  |  |  |
| **LOS_2** |  |  | 0.816 |  |  |
| **LOS_3** |  |  | 0.876 |  |  |
| **LOS_4** |  |  | 0.716 |  |  |
| **DTSQ_1** |  | 0.747 |  |  |  |
| **DTSQ_2** |  |  |  |  | 0.891 |
| **DTSQ_4** |  |  |  |  | 0.885 |
| Extraction Method: Principal Axis Factoring.   Rotation Method: Promax with Kaiser Normalization. | | | | | |
| a. Rotation converged in 6 iterations. | | | | | |
