# Supplementary material for: Coping with diabetes: Provider attributes that influence type 2 diabetes adherence
Source: PLoS One. 2019 Apr 2;14(4):e0214713. doi: 10.1371/journal.pone.0214713 (PMC6445439; doi:10.1371/journal.pone.0214713)
Supplement: S2 Table — (DOCX) [file pone.0214713.s010.docx]

# **S2 Table. Factor correlation matrix**

| **Construct** | **Compassion** | **Self-Mgt** | **Optimism** | **Coping Ability** | **Treat. Sat.** |
| --- | --- | --- | --- | --- | --- |
| **Compassion** | 1.000 | 0.279 | 0.688 | 0.694 | 0.254 |
| **Self-Mgt** | 0.279 | 1.000 | 0.324 | 0.290 | 0.382 |
| **Optimism** | 0.688 | 0.324 | 1.000 | 0.696 | 0.260 |
| **Coping Ability** | 0.694 | 0.290 | 0.696 | 1.000 | 0.263 |
| **Treatment Sat.** | 0.254 | 0.382 | 0.260 | 0.263 | 1.000 |
| Extraction Method: Principal Axis Factoring.   Rotation Method: Promax with Kaiser Normalization. | | | | | |
