# Supplementary material for: Coping with diabetes: Provider attributes that influence type 2 diabetes adherence
Source: PLoS One. 2019 Apr 2;14(4):e0214713. doi: 10.1371/journal.pone.0214713 (PMC6445439; doi:10.1371/journal.pone.0214713)
Supplement: S3 Table — (DOCX) [file pone.0214713.s011.docx]

| **S3 Table. One-way ANOVA between groups** | | | | | | |
| --- | --- | --- | --- | --- | --- | --- |
|  | | Sum of Squares | df | Mean Square | F | Sig. |
| DSMQ_1 | Between Groups | 13.854 | 2 | 6.927 | 4.665 | .010 |
| DSMQ_2 | Between Groups | 4.938 | 2 | 2.469 | 3.870 | .022 |
| DSMQ_3 | Between Groups | 10.294 | 2 | 5.147 | 3.514 | .031 |
| DSMQ_4 | Between Groups | 12.121 | 2 | 6.061 | 3.978 | .019 |
| DSMQ_5 | Between Groups | 19.408 | 2 | 9.704 | 7.524 | .001 |
| DTSQ_1 | Between Groups | 8.990 | 2 | 4.495 | 5.035 | .007 |
| DTSQ_2 | Between Groups | 15.511 | 2 | 7.756 | 6.838 | .001 |
| DTSQ_4 | Between Groups | 15.556 | 2 | 7.778 | 10.273 | .000 |

**DSMQ= Diabetes Self-Management Questionnaire Scale; DTSQ= Diabetes Treatment Satisfaction Questionnaire*
