# Supplementary material for: Coping with diabetes: Provider attributes that influence type 2 diabetes adherence
Source: PLoS One. 2019 Apr 2;14(4):e0214713. doi: 10.1371/journal.pone.0214713 (PMC6445439; doi:10.1371/journal.pone.0214713)
Supplement: S4 Table — (DOCX) [file pone.0214713.s012.docx]

# **S4 Table. Comparison of group means**

| Demographic Sample Characteristics | | (I) Group | (J) Group | Mean Diff (I-J) | Std. Error | Sig. |
| --- | --- | --- | --- | --- | --- | --- |
|  |  |  |  |  |  |  |
| DSMQ_1 | Tukey HSD | 1 | 2 | .614^*^ | .201 | .007 |
|  |  |  | 3 | .193 | .134 | .324 |
|  |  | 2 | 1 | -.614^*^ | .201 | .007 |
|  |  |  | 3 | -.421 | .184 | .058 |
|  |  | 3 | 1 | -.193 | .134 | .324 |
|  |  |  | 2 | .421 | .184 | .058 |
| DSMQ_2 | Tukey HSD | 1 | 2 | .207 | .132 | .259 |
|  |  |  | 3 | -.116 | .088 | .384 |
|  |  | 2 | 1 | -.207 | .132 | .259 |
|  |  |  | 3 | -.323^*^ | .120 | .020 |
|  |  | 3 | 1 | .116 | .088 | .384 |
|  |  |  | 2 | .323^*^ | .120 | .020 |
| DSMQ_3 | Tukey HSD | 1 | 2 | .509^*^ | .198 | .028 |
|  |  |  | 3 | .236 | .133 | .181 |
|  |  | 2 | 1 | -.509^*^ | .198 | .028 |
|  |  |  | 3 | -.273 | .181 | .287 |
|  |  | 3 | 1 | -.236 | .133 | .181 |
|  |  |  | 2 | .273 | .181 | .287 |
| DSMQ_4 | Tukey HSD | 1 | 2 | .313 | .202 | .270 |
|  |  |  | 3 | .382^*^ | .136 | .014 |
|  |  | 2 | 1 | -.313 | .202 | .270 |
|  |  |  | 3 | .069 | .184 | .926 |
|  |  | 3 | 1 | -.382^*^ | .136 | .014 |
|  |  |  | 2 | -.069 | .184 | .926 |
| DSMQ_5 | Tukey HSD | 1 | 2 | .072 | .186 | .920 |
|  |  |  | 3 | .451^*^ | .125 | .001 |
|  |  | 2 | 1 | -.072 | .186 | .920 |
|  |  |  | 3 | .379 | .170 | .067 |
|  |  | 3 | 1 | -.451^*^ | .125 | .001 |
|  |  |  | 2 | -.379 | .170 | .067 |
| DTSQ_1 | Tukey HSD | 1 | 2 | .257 | .159 | .241 |
|  |  |  | 3 | -.179 | .104 | .200 |
|  |  | 2 | 1 | -.257 | .159 | .241 |
|  |  |  | 3 | -.435^*^ | .146 | .008 |
|  |  | 3 | 1 | .179 | .104 | .200 |
|  |  |  | 2 | .435^*^ | .146 | .008 |
| DTSQ_2 | Tukey HSD | 1 | 2 | .623^*^ | .179 | .002 |
|  |  |  | 3 | .051 | .117 | .900 |
|  |  | 2 | 1 | -.623^*^ | .179 | .002 |
|  |  |  | 3 | -.572^*^ | .164 | .002 |
|  |  | 3 | 1 | -.051 | .117 | .900 |
|  |  |  | 2 | .572^*^ | .164 | .002 |
| DTSQ_4 | Tukey HSD | 1 | 2 | .613^*^ | .146 | .000 |
|  |  |  | 3 | .032 | .096 | .941 |
|  |  | 2 | 1 | -.613^*^ | .146 | .000 |
|  |  |  | 3 | -.582^*^ | .134 | .000 |
|  |  | 3 | 1 | -.032 | .096 | .941 |
|  |  |  | 2 | .582^*^ | .134 | .000 |

*****Group 1= Amazon Mechanical Turk; Group 2= Facebook diabetes social support group; and 3= Qualtrics****.*
